# An Individualized, Less-Invasive Surgical Approach Algorithm Improves Outcome in Elderly Patients Undergoing Mitral Valve Surgery

**DOI:** 10.3390/jcdd10010028

**Published:** 2023-01-11

**Authors:** Ulvi Cenk Oezpeker, Fabian Barbieri, Daniel Höfer, Can Gollmann-Tepeköylü, Johannes Holfeld, Florian Sommerauer, Julian Wagner, Sasa Rajsic, Suat Ersahin, Nikolaos Bonaros, Michael Grimm, Müller Ludwig

**Affiliations:** 1 Department of Cardiac Surgery, Medical University of Innsbruck, 6020 Innsbruck, Austria; 2 Department of Cardiology, Charité—Universität zu Berlin, Hindenburgdamm 30, 12203 Berlin, Germany; 3 Department of Anesthesiology and Intensive Care Medicine, Medical University of Innsbruck, 6020 Innsbruck, Austria; 4 Sakarya Eğitim ve Araştırma Hospital, 54100 Adapazarı, Turkey

**Keywords:** elderly patients, mitral valve surgery, less invasive, surgical trauma reduction, mitral valve repair

## Abstract

Background: For mitral valve surgery (MVS) in elderly, frail patients with increasing life expectancy, finding the least harmful means of access is a challenge. In the complexity of MVS approach evolution, using three different approaches (mini-thoracotomy (MT), partial upper-sternotomy (PS), full-sternotomy (FS), we developed a personalized, minimized-invasiveness algorithm for MVS. Methods: In this retrospective analysis, 517 elderly patients (≥70 years) were identified who had undergone MVS ± TV repair. MVS was performed via MT (*n* = 274), FS (*n* = 128) and PS (*n* = 115). The appropriate access type was defined according to several clinical patient conditions. Using uni- and multivariate regression models, we analyzed combined operative success (residual MV regurgitation, conversion to MV replacement or larger thoracic incisions); perioperative success (30-days mortality, thoracotomy, ECMO, pacemaker implantation, dialysis, longer ventilation); and reoperation-free long-term survival. An additional EuroSCORE2 adjustment was performed to reduce the bias of clinical conditions between all access types. Results: The EuroSCORE2-adjusted Cox regression analysis showed significantly increased reoperation-free survival in the MT cohort compared to FS (HR 0.640; 95% CI 0.442–0.926; *p* = 0.018). Mortality was additionally reduced after the implementation of PS (*p* = 0.023). Combined operative success was comparable between the three access types. The perioperative success was higher in the MT cohort compared to FS (OR 2.19, 95% CI 1.32–3.63; *p* = 0.002). Conclusion: Less-invasive approaches in elderly patients improve perioperative success and reoperation-free survival in those undergoing MVS procedures.

## 1. Introduction

Mitral valve (MV) disease is common in elderly patients and occurs at an increasing frequency with advanced age [1]. However, choosing between conservative, transcatheter and surgical treatment is an individualized process dependent on several factors, which modify morbidity and mortality risk [2,3,4]. Most critical parameters include age, comorbidities, time point of surgical intervention and possibility of repairing the MV pathology [4,5,6]. Due to prolonged life expectancy and the accompanying increase in frailty and concomitant heart failure of elderly patients, surgeons need to seek alternative, reproducible and less harmful algorithms to reduce operative trauma and mortality. An increase in minimally invasive heart surgery has been observed in recent decades [7]. Most of the publications about minimally invasive mitral valve surgery (MIMVS) in the elderly focus on low-risk degenerative MV etiologies [6,8,9]. However, despite the increasing adoption of MIMVS, patients with absolute or relative contraindications for MIMVS exist in daily routine cardiac surgery [10,11]. These certain clinical conditions are potentially harmful limitations for safe MVS via the right MT access type, making FS the sole, alternatively preferred, safe and quick access type in 45% of MV surgeries in almost all German centers [7,12]. Moreover, longer operative times and learning curves associated with the use of long-shafted instruments and endoscopic techniques still prevent the broad acceptance of less-invasive procedures [13]. In addition, MV repair rates in complex pathologies are generally based on institutional experience and may additionally limit the number of patients suitable for MIMVS via MT [14]. However, the benefits of MIMVS in elderly patients and data on transcatheter interventions are inconclusive. Some investigations have demonstrated that MIMVS reduces operative trauma, with long-term survival at five and seven years of 55% and 52%, respectively [8,9,15]. Patients who are considered unsuitable for MT undergo operation with conventional FS. Based on more than 20 years of experience in minimally invasive cardiac procedures, including PS and MT for aortic valve replacement and more than 1000 video-assisted or fully endoscopic MV procedures via MT, we adapted partial upper sternotomy as a complementary, less-invasive mode of access for MVS [16]. In the complexity of MVS approach evolution over the decades, we aimed to keep MV repair rates high and chose a tailored approach for MVS, in order to minimize mortality in elderly patients. In this study, we not only focused on MIMVS in elderly patients with low-risk degenerative MV pathologies, but we also tried to include all higher risk heterogenous MV etiologies and patients with contraindications for MT by implementing a personalized and less-invasive algorithm.

## 2. Methods

### 2.1. Study Design and Patients

The data for this investigation were obtained from the MVS database of the Department of Cardiac Surgery of the Medical University of Innsbruck, Austria. Survival data were acquired by the national death registry of Austria (Statistik Austria). Patients without an event were censored at the end of the follow up. The follow up was conducted by outpatient visits as well as phone calls to the patients and their attending physicians, who sent us the echocardiographic and ECG findings. Written informed consent for the scientific use of clinical data was obtained from all patients as part of the quality control program of the Medical University of Innsbruck, which was approved by the local ethics committee (13 February 2020; EC Nr.: 1203/2019) and the Austrian Ministry of Health. The investigation complied with the principles outlined in the Declaration of Helsinki.

In this longitudinal retrospective cohort study, in our center, we reviewed the records of 1534 patients who had undergone isolated MVS or combined with TV repair ± atrial ablation procedures, without coronary artery bypass grafting and aortic valve replacement, between March 2001 and February 2021. Patients with age < 70 years; redo surgery; urgent or salvage surgery with, for example, active endocarditis; and concomitant surgery of the ascending aorta were excluded. Eight patients were excluded due to incomplete data, leaving 517 patients who were finally included in the data analysis. For the analysis, the patients were divided into three groups depending on the surgical approach (MT *n* = 274; FS *n* = 128; PS *n* = 115).

**Figure 1:**
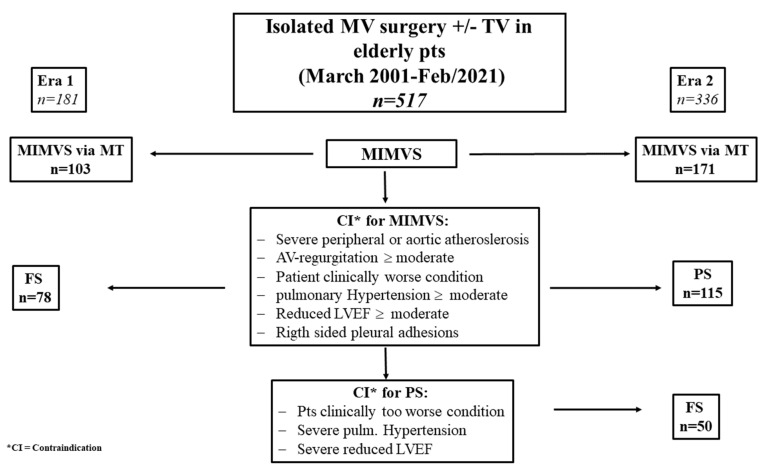
Flowchart for the algorithm of appropriate approach in elderly patients. * CI = contraindication, ± = with or without.

Patient allocation to the adequate approach was dictated by institutional protocols (Table 1, Figure 1). Concomitant cardiac surgery, other than TV repair or atrial ablation procedures; aortic valve regurgitation with a possible risk of aortic valve replacement; severe right-sided pulmonary adhesions; significant mitral annulus calcification with the need for unitary decalcification; severely atherosclerotic aorta descendens or femoral arteries not amenable for peripheral cannulation, and a dilated ascending aorta > 45 mm; systolic pulmonary pressure > 50 mm Hg; and severely impaired left-ventricular function were disregarded as ideal candidates for MT access.

Based on this certain risk profile, MVS was conducted via FS instead of MT up to 2011 (era 1). In 2011, the implementation of PS access started as a complementary, less-invasive access type for patients with clinical contraindications for MT (era 2).

### 2.2. Surgical Procedures

The institutional operative techniques have been described in detail for all three access types in earlier publications [16,17].

The PS approach with extension of the transseptal incision into the atrial roof was described in detail by Gillinov et al., and Svensson et al. Briefly, we first performed a PS starting at the sternal notch with extension into the fourth intercostal space to the left side. Cannulation for the cardiopulmonary bypass (CPB) was performed via the ascending aorta; the superior vena cava was cannulated directly, while the inferior vena cava (IVC) was cannulated percutaneously or surgically via the femoral vein using Seldinger’s technique. Correct positioning was achieved after establishing CPB and the superior and IVC were snared in order to prevent air-lock. After cross-clamping of the ascending aorta cardioplegia was first applied antegrade and, after opening the right atrium, repeated retrograde to the coronary sinus under direct visualization via a catheter. Afterwards the incision of the interatrial septum at the level of the fossa ovalis was performed. This cut was prolonged to the right atrial incision and extended along the roof of the left atrium towards the aorta. With this technique, excellent surgical exposure was achieved. 

In the FS access type, the cannulation was similar to the PS approach. The direct cannulation of the IVC was performed. Access to the MV was performed via the dissection of the interatrial groove in every case, independently according to whether a tricuspid valve repair with opening of the right atrium was performed or not.

In the MT cohort, cardiopulmonary bypass was installed via femoro-femoral cannulation with an additional distal leg perfusion to avoid leg perfusion issues. An additional venous cannula was inserted into the right jugular vein in case of TVR or patients with increased body surface area (BSA) for optimal drainage. The MT access was performed through the fourth intercostal space via periareolar or a 3 cm long skin cut lateral to the nipple and a similar incision in the submammary fold was performed depending on a male or female patient. The third intercostal space on the anterior axillary line was used for the scope and the Chitwood clamp. The soft tissue retractor Alexis wound protector was used to avoid rib spreading.

Common mitral-repair techniques, including chordal replacement (single polytetrafluoroethylene (PTFE) chords, secondary chords or pre-fabricated PTFE loops), leaflet resection, sliding plasty or indentation closure, were applied. A semi-rigid annuloplasty ring was used in all procedures. Moreover, a tricuspid valve repair was performed in all patients with severe tricuspid valve regurgitation or annular dilatation above 21 mm/m^2^ BSA.

### 2.3. Definitions

The major outcome parameter was reoperation-free survival defined as freedom from death and reoperation during follow up, due to valve-related complications (native valve-related: new onset of MV-regurgitation > moderate or prosthetic valve-related: paravalvular leakage, valve degeneration, valve thrombosis, endocarditis).

Further major outcome parameters were operative and perioperative success as composite endpoints within the first 30 days. Combined operative success was defined as freedom of death, successful primary MV repair without conversion to replacement or to larger thoracic incisions, and residual mitral regurgitation ≥ moderate or prosthetic valve-related paravalvular leakage.

The definition for combined perioperative success was 30-days survival, freedom from perioperative myocardial infarction (Fourth Universal Definition of Myocardial infarction), stroke, extracorporeal membrane oxygenation support, renal failure necessitating dialysis, persistent pacemaker implantation, mechanical ventilation > 24 h and re-operation due to any reason (including bleeding).

The subgroup analyses focused on long-term outcome according to three distinct age classes (70–74, 75–79 and ≥80 years of age), the comparison of MV repair versus replacement, and the comparison of reoperation-free survival between degenerative and secondary MV etiologies.

## 3. Statistical Analysis

Categorical variables are displayed as absolute numbers and percentages, and continuous variables as median and their respective 25th and 75th percentile. The distribution of continuous variables was assessed by an inspection of the histograms and use of the Shapiro-Wilk test. Group-specific differences were analyzed either by an ANOVA or Kruskal-Wallis test for continuous variables, according to their distribution, and by the chi-square test for categorical variables. To estimate the group-specific differences for operative and perioperative success, as well as their components, binary logistic regression models were calculated. The weight of factors limiting long-term survival and reoperation-free survival were calculated by applying Cox regression models. These models were, if indicated, also adjusted for the EuroSCORE2. To determine the potential advantages of the less-invasive methods, FS was used as a reference cohort in all models. A univariate subgroup analysis was calculated by creating Kaplan–Meier curves, and differences were assessed by the log-rank test. The analysis was conducted using IBM SPSS, version 24 (IBM Corporation, Armonk, NY, USA), and graphics were designed using GraphPad PRISM, version 5 (GraphPad Software, Inc., La Jolla, CA, USA). *p*-values of 0.05 or less were considered statistically significant.

## 4. Results

### 4.1. Baseline Characteristics and Intraoperative Parameters

Baseline and intraoperative characteristics are illustrated in Table 2, Table 3 and Table 4. For most variables, all the given preoperative data show statistically significant highest morbidity in the PS cohort and lowest in the MT cohort. The EuroSCORE2 was found to be highest in the FS group (*p* < 0.001), while the pre-operative NT-proBNP levels were increased in the PS cohort (*p* = 0.004). Patients requiring dialysis before surgery were only present in the PS cohort (*p* = 0.030).

Regarding etiology, there was no significant difference in the incidence of MV pathology (*p* = 0.163). Overall, the MV repair rate was 74.1% (*n* = 383). The highest rates of MV repair (87.6%, *n* = 240, *p* = < 0.001) and partial additional TV repair (34.3%, *n* = 94, *p* = 0.005) were seen in the MT cohort. In addition, CPB times (198 (158–232) min, *p* < 0.001) and cross-clamp times (106 (84–126) min, *p* = 0.004) were significantly longer in the MT cohort. Comparable frequencies were found for moderate-to-severe annulus calcification in both sternotomy cohorts (32.2% vs. 38.3%), but significantly less in the MT cohort (0.7% *p* < 0.001).

### 4.2. Long-Term Outcome

**Figure 2:**
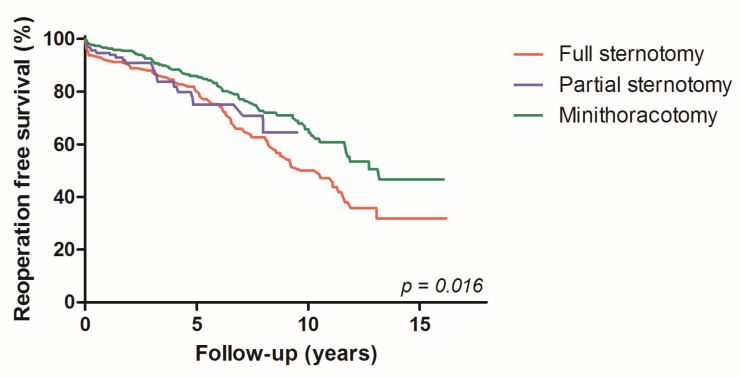
Kaplan-Meier curves: reoperation-free survival, according to the three access types.

The total median follow up was 5.6 (IQR 2.7–8.7) years. All-cause mortality was 28.7% (*n* = 147 patients) during this period, while three patients (0.6%) required mitral valve related re-operation. During long-term follow up, 10-year reoperation-free survival was achieved in 65.7% of patients for MT, 64.4 % for PS and 50.2% for FS, while univariate comparison using overall follow up reached statistical significance (*p* = 0.016) (Figure 2). The EuroSCORE2-adjusted Cox regression analysis showed comparable mortality hazard ratios for FS and PS (HR 0.891; 95% CI 0.533–1.490; *p* = 0.660), whereas MT resulted in a significantly lower mortality hazard compared to FS (HR 0.640; 95% CI 0.442–0.926; *p* = 0.018).

### 4.3. Operative and Perioperative Success

The combined operative success rate was given for all three access types as well as in the EuroSCORE2-adjusted analysis, and it showed no statistically significant difference.

The combined perioperative success rate reached statistical significance with the MT-access and was higher after EuroSCORE2 adjustment (OR 2.19, 95% CI 1.32–3.63; *p* = 0.002) compared to FS. The six-fold higher risk of 30-days mortality was seen in the FS-cohort compared with the MT access type (OR 6.69; 95% CI 1.33–33.61; *p* = 0.021). An advantage of MT compared to FS was also seen in longer ventilation times (OR 2.17; 95% CI 1.23–3.84; *p* = 0.007) and a lower incidence of PM implantations (OR 15.79; 95% CI 1.9–129.8; *p* = 0.01). In addition, more patients required renal replacement therapy in the FS-cohort compared to MT (OR 11.99; 95% CI 3.41–42.23; *p* < 0.001). However, there was no statistical difference between FS and PS (Table 5).

**Figure 3:**
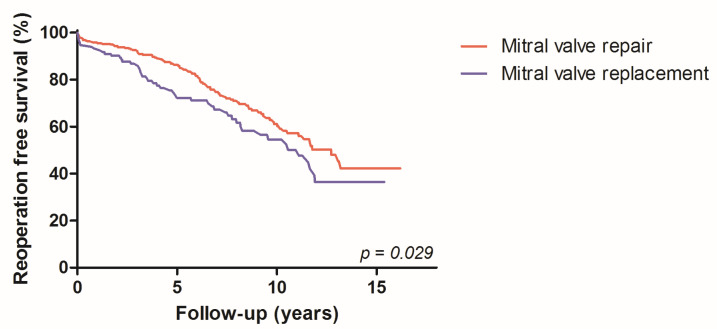
Kaplan–Meier curves: reoperation-free survival benefit of MV repair.

In the subgroup analysis, the univariate analyses showed similar reoperation-free long-term survival between primary and secondary MV pathologies (*p* = 0.187). This similarity when comparing the results was also found if the cohorts were broken down into the three access types (FS *p* = 0.220; PS *p* = 0.877; MT *p* = 0.253). In addition, the log-rank test showed a reoperation-free survival benefit of MV repairs (63.6%) over MV replacement (57.8%, *p* = 0.030) (Figure 3).

**Figure 4:**
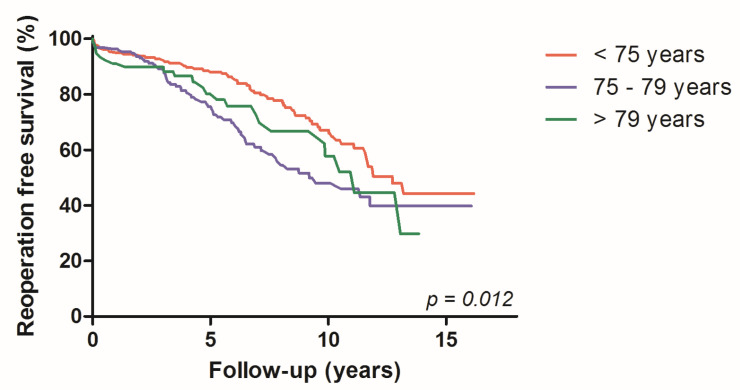
Kaplan-Meier curves: reoperation free survival, according to age groups.

Moreover, the highest 10-year reoperation-free survival rate was observed for the age group 70–74 years (70.3%), decreasing for 75–79 years (60.2%) and being lowest for patients ≥ 80 years (55.8%, *p* = 0.012) (Figure 4). In addition, the long-term mortality was significantly reduced between era 1 and era 2 in this high-risk patient cohort (log rank *p* = 0.023).

## 5. Discussion

Safe and reproducible MVS in elderly patients, given their frailty and higher incidence of comorbidities such as heart failure, is a growing challenge due to prolonged life expectancy. Our investigation described the short-term and long-term benefits of an individualized, least-possible invasive surgical algorithm isolated MVS or combined with TVS, in this population. It has to be pointed out that this investigation is not a comparison of three different access types, as MT was the preferred approach in our center. It is an algorithm to reduce the operative trauma in these frail patients to find the least-harmful intervention.

Several studies have described the superiority of MIMVS compared to FS, due to a reduction in operative trauma, especially in elderly patients. However, data on MIMVS either mainly focus on low-risk degenerative MV diseases with high MV repair rates [8,9,15] or, on secondary MV etiologies. As elderly patients who are frail and have accompanying heart failure more often have complex MV pathologies, MV repairs can sometimes be extremely difficult and harmful, pushing many surgeons to opt for straightforward MV replacement or even reject surgery altogether. However, several studies described the superiority of MV repair over replacement, especially regarding survival [18]. Our data are in accordance with these investigations and support the principal strategy to repair the valve whenever possible [17,19], independent of access and complexity. Our data describe an overall figure of 74.1% of MV repair rates, which is higher compared to the study by Seeburger et al. [6], despite including all MV pathologies. In addition, our univariate analyses on reoperation-free long-term survival were similar between primary and secondary MV pathologies; therefore we decided to analyze all MV pathologies together. Our patient cohort included degenerative MV diseases, as well as patients with mitral annular calcifications (17%, *n* = 88), MV stenosis (8.1% *n* = 42), inactive endocarditis (3.1%, *n* = 16) and Barlow disease, which are generally viewed as complex indications for MV repair, especially for less-invasive approaches. Most patients with successful MV repair rates were found in the MT cohort (87%), which reflects the increased survival, safety and reproducibility, even in complex pathologies with this access, despite longer X-clamp and CPB times. It has to be pointed out that the MT cohort contained two patients with peripheral arterial occlusive disease, which is a contraindication for this approach. In these two patients, the right carotid artery was used for arterial cannulation for extracorporeal circulation [20]. However, the data for the two other access types showed similar results, underlining the safety and reproducibility of PS access.

Our data describe the lowest comorbidities, comparable operative but statistically significant highest perioperative success and consecutive 10-year reoperation-free survival rates in the MT cohort, which can be explained by healthier patients and the highest amount of less-operative trauma in this cohort. Overall, the probability of 10-year reoperation-free survival was 71% in our investigation, which is higher than the previous published data, which mainly exclude complex MV pathologies. These data reveal almost equal life expectancy in this cohort compared to the general population of patients, as indicated by Statistik Austria (URL: www.statistik.at), accessed on 1 November 2022. However, selection bias might be the most important factor for outcome. Yet, multivariate analyses with EuroSCORE2 adjustment were applied to reduce this bias, and similar results were obtained. These results are in accordance with the publication of Al Otaibi et al. [21].

Due to limitations for MIMVS, which do exist in daily cardiac surgery [13], FS is the preferred alternative bail-out strategy in almost all centers worldwide. In 47% of the patients in our study cohort, certain clinical or anatomical conditions were considered as contraindications for MT due to the high risk of complications and mortality [10]. We adopted our personalized, least-invasive access strategy with the implementation of PS 2011, resulting in a reduction in the long-term mortality between era 1 and era 2 in this patient cohort. PS was associated with lower postoperative complication rates and survival benefit compared to FS in a previous publication [22], yet this remains unproven in the elderly patient cohort. Nevertheless, the bias of changing surgical and intensive care unit strategies over 20 years may not be excluded by our data, leaving a lack of conclusive evidence for improved outcomes with lower invasiveness.

Despite the high rates of ECMO implantation within the MT access group, the incidence of low cardiac output syndrome was detected most often in the FS and lowest in the MT cohort. This benefit may be explained by the partial integrity of the pericardium in the minimally or less-invasive access types [23,24]. In addition, the regression analyses displayed a lower incidence of prolonged ventilation, which supports the result of several studies, and postoperative new onset of renal replacement therapy in the MT-cohort [25,26]. All these factors together facilitate faster mobilization in elderly patients, which might have an impact on short- and long-term outcomes. Robotic cardiac surgery would probably further reduce operative trauma and improve survival [27].

One of the crucial aspects of our study was that the preoperative data of the PS cohort revealed higher morbidity, but similar perioperative and operative success and long-time survival compared to FS. The reason for the lack of statistical differences between PS over FS can be explained by the low statistical power and the shorter follow-up times in era 2 of these access types.

However, in 5% of the patients, conversion to FS was necessary. In the MT cohort, conversion to FS was necessary due pericardial adhesions *n* = 4, uncontrollable bleeding *n* = 3, pleural adhesions *n* = 2 and limited surgical exposure *n* = 2. In the PS cohort, the reasons for conversion were pericardial adhesions (*n* = 7), uncontrollable bleeding (*n* = 5, atrioventricular dissection after en bloc decalcification of the mitral annulus) and surgical interventions (*n* = 2, additionally, CABG to the RCX due to cardiac ischemia and ascending aortic replacement due to dissection). Limited exposure was seen in this cohort in one patient; however, the exposure was also very difficult after FS.

Further analysis revealed a lower incidence of PM implantation in the MT cohort and highest in the PS cohort; the cohort might also have an impact on mortality rates. The higher PM implantation rates may be explained by the fact that, for PS access, an extension of the trans-septal incision—which is a subject of controversial discussion [22,28,29]—into the left atrial roof was performed, while for MT and FS, a transatrial or transeptal approach without extension was performed for the exploration of the MV. However, a statistically significant difference was found only between MT and FS.

### Limitations

One major limitation of this investigation was the adjudication of patients to one or the other approach with marked preference for MT, potentially creating an allocation bias. With the continuous evolution of surgical techniques and growing experience with MIMVS, the relative contraindications for the MT approach have changed dramatically, leaving only a few indications for PS, and even fewer for FS in recent years. Furthermore, the changing intensive-care therapeutic strategies might have an impact on better outcomes, which cannot be excluded by our investigation.

Further limitations were the retrospective data analysis of a single center experience and the relatively small sample size. Therefore, we may not be able to rule out potential underpowering for the assessment of differences between study groups with regard to the outcome parameters.

In conclusion, the presented results demonstrate that the individualized, least-invasive possible access type with reduced trauma, in combination with the principal intention to repair the MV, reduced mortalities in this high-risk cohort. All minimally invasive or less-invasive access types are safe and reproducible MVS approaches. High rates of MV repair and reduced operative trauma permit short and long-term benefits in elderly patients.

## Figures and Tables

**Table 1:** Reasons against MT and PS access.

	Era 1 *n* = 181	Era 2 *n* = 336	
	FS Instead of MT *n* = 78	PS Instead of MT *n* = 115	FS Instead of MT *n* = 50
Severe peripheral or aortic atherosclerosis (%, *n*)	3.3 (6)	20.9 (24)	3.3 (6)
AV regurgitation ≤ moderate (%, *n*)	3.9 (10)	7.8 (9)	3.9 (10)
Patient in a clinically worse condition (%, *n*)	11.6 (21)	16.5 (19)	11.6 (21)
Moderate-to-major AC (%, *n*)	9.9 (18)	34 (34)	9.9 (18)
Pulmonary hypertension ≥ moderate (%, *n*)	6.6 (12)	18.3 (21)	6.6 (12)
Reduced LVEF ≥ moderate	5.0 (9)	4.3 (5)	5.0 (9)
Right-sided pleural adhesions (%, *n*)	1.1 (2)	1.7 (2)	1.1 (2)
Surgical training reasons (%, *n*)	0	0.9 (1)	0

Abbreviations: AC = annulus calcification; AV = aortic valve; FS = full sternotomy; MT = mini-thoracotomy, PS = partial sternotomy.

**Table 2:** Baseline characteristics of the study cohort.

	MVS (Total) *n* = 517	MT-MVS *n* = 274	PS-MVS *n* = 115	FS-MVS *n* = 128	*p*-Value
Age (years) ^1^	75 (72–78)	74 (63–77)	76 (72–79)	75 (72–79)	0.006
Gender, females (%, *n*)	56.5 (292)	52.6 (144)	58.3 (67)	62.5(80)	0.154
Primary MV disease (%, *n*)	82.6 (427)	79.9 (219)	87.8 (101)	83.6 (107)	0.163
BSA (m^2^)	1.80 (1.66–1.94)	1.80 (1.70–2.0)	1.77 (1.62–1.94)	1.74 (1.63–1.87)	<0.001
DM (%, *n*)	36.2 (187)	52.9 (145)	18.3 (21)	16.4 (21)	<0.001
IDDM (%, *n*)	4.6 (24)	6.2 (17)	2.6 (3)	3.1 (4)	0.191
Art.hypertension (%, *n*)	65.8 (340)	52.9 (145)	81.7 (94)	78.9 (101)	<0.001
COPD ≥ GOLD 2 (%, *n*)	18.4 (95)	30.5 (51)	35.4 (58)	30.5 (50)	<0.001
PAOD (%, *n*)	2.3 (12)	0.7 (2)	6.1 (7)	2.3 (3)	0.006
Dialysis (%, *n*)	0.4 (2)	0	1.7 (2)	0	0.030
Smoking history (%, *n*)	9.5 (49)	5.1 (14)	16.5 (19)	12.5 (16)	<0.001
HLP (%, *n*)	39.1 (202)	28.8 (79)	53.9 (62)	47.7 (61)	<0.001
Prev.CVE (%, *n*)	4.1 (21)	1.5 (4)	7 (8)	7 (9)	0.006
EuroSCORE2 (%) ^1^	3.10 (1.80–4.73)	2.20 (1.31–3.70)	3.70 (2.51–5.20)	4.21 (3.15–6.43)	<0.001
LV-EF (%) ^1^	60 (51–64)	60 (52–64)	58 (50–65)	57 (50–63)	0.114
NYHA III (%, *n*)	56.1 (290)	51.8 (142)	61.7 (71)	60.2 (77)	0.112
NYHA IV (%, *n*)	5.0 (26)	3.3 (9)	8.7 (10)	5.5 (7)	0.081
i-Afib (%, *n*)	42.4 (219)	37.2 (102)	43.5 (50)	52.3 (67)	0.016
p-Afib (%, *n*)	15.3 (79)	9.5 (26)	25.2 (29)	21.1 (27)	<0.001
sPAP > 55 mmHg (%, *n*)	15.3 (79)	5.1 (14)	32.1 (34)	32 (31)	<0.001
NT-proBNP (ng/l) ^1^	1218 (550–2133)	1148 (472–1800)	1410 (705–2979)	1220 (723–2431)	0.004

Abbreviations: BSA = body surface area; COPD = chronic obstructive pulmonary disease; CVE = cerebrovascular event; DM = diabetes mellitus; FS = full sternotomy; HLP = hyperlipidemia; IDDM = insulin-dependent diabetes mellitus; i-Afib = intermittent atrial fibrillation; LV-EF = left-ventricular ejection fraction; MT = mini thoracotomy, MV = mitral valve; MVS = mitral valve surgery; p-Afib = permanent atrial fibrillation; PAOD = peripheral arterial occlusive disease; PS = partial upper sternotomy; sPAP = systolic pulmonary artery pressure; ^1^ continuous variables are expressed as median and interquartile range.

**Table 3:** Secondary intraoperative outcomes.

Intraoperative Outcomes	MVS (Total) *n* = 517	MT-MVS *n* = 274	PS-MVS *n* = 115	FS-MVS *n* = 128	*p*-Value
MV repair (%, *n*)	74.1 (383)	88.97 (242)	62.6 (72)	53.9 (69)	<0.001
Switch MV repair to replacement (intraoperatively) (%, *n*)	2.7 (14)	1.5 (4)	2.6 (3)	5.5 (7)	0.070
Additional TV repair (%, *n*)	40.8 (211)	34.3 (94)	46.1 (53)	50 (64)	0.005
Ablation surgical (%, *n*)	15.3 (79)	17.5 (48)	9.6 (11)	15.6 (20)	0.137
Cardiopulmonary bypass time (min) ^1^	168 (137–211)	198 (158–232)	151 (130–176)	144 (113–170)	<0.001
Aortic cross-clamp time (min) ^1^	101 (80–123)	106 (84–126)	94 (83–115)	93 (71–118)	0.004
Conversion to FS (%, *n*)	5.0 (26)	4 (11)	13 (15)	0	<0.001
Second pump run/X-clamp (%, *n*)	4.3 (22)	3.0 (8)	6.1 (7)	5.5 (7)	0.365
Moderate-to-major annulus calcifications (%, *n*)	17.0 (88)	0.7 (2)	32.2 (37)	38.3 (49)	<0.001
En bloc decalcifications *(%*, *n)*	2.9 (15)	0.7 (2)	3.5 (4)	7.0 (9)	0.002

Abbreviations: FS = full sternotomy; MVS = mitral valve surgery; MT = mini thoracotomy PS = partial upper sternotomy; ^1^ continuous variables are expressed as median and interquartile range.

**Table 4:** Secondary postoperative outcomes.

Postoperative Outcomes	MVS (Total) *n* = 517	MT-MVS *n* = 274	PS-MVS *n* = 115	FS-MVS *n* = 128	*p*-Value
MV regurgitation ≥ 2 after MV-reapair * (%, *n*)	4.2 (16)	2.9 (7)	1.4 (1)	11.6 (8)	0.040
Mild PVL * (in the MV-replacement group) (%, *n*)	2.2 (3)	0 (0)	4.7 (2)	1.7 (1)	0.113
30-days mortality (%, *n*)	2.1 (11)	0.7 (2)	2.6 (3)	4.7 (6)	0.035
1-year mortality (%, *n*)	4.5 (23)	2.2 (6)	5.2 (6)	8.6 (11)	0.016
Extracorporeal membrane oxygenation, (%, *n*)	2.5 (13)	3.3 (9)	2.6 (3)	0.8 (1)	0.327
Cardiac low-output syndrome, (%, *n*)	10.4 (54)	3.3 (9)	14.8 (17)	21.9 (28)	<0.001
Tamponade or excessive bleeding (%, *n*)	6.2 (32)	5.5 (15)	7.8 (9)	6.3 (8)	0.680
Hemofiltration/-dialysis (%, *n*)	7.9 (41)	1.1 (3)	20 (23)	11.7 (15)	<0.001
Ventilation >24 hrs (%, *n*)	15.3 (79)	10.9 (30)	19.1 (22)	21.1 (27)	0.013
Red blood units (total) ^1^	1 (1–2)	0 (0–2)	1 (1–3)	1 (1–3)	<0.001
Intensive care unit length (days) ^1^	1 (1–2)	1 (1–1)	1 (1–4)	2 (1–9)	<0.001
Hospital stay (days) ^1^	8 (7–11)	8 (7–9)	8 (7–12)	10 (8–12)	<0.001
Deep wound infection (%, *n*)	1.4 (7)	0	1.7 (2)	3.9 (5)	0.006
Cerebrovascular adverse event (%, *n*)	0.8 (4)	0	0.9 (1)	2.3 (3)	0.044
Pacemaker implantation (%, *n*)	3.4 (18)	0.4 (1)	8.7 (10)	5.5 (7)	<0.001
Myocardial infarction (%, *n*)	0.4 (2)	0	0.9 (1)	0.8 (1)	0.648

Abbreviations: FS = full sternotomy; MVS = mitral valve surgery; MT = mini thoracotomy; PS = partial upper sternotomy; PVL = paravalvular leakage; ^1^ continuous variables are expressed as median and interquartile range; * at 30 days follow up.

**Table 5:** Operative and perioperative (30-day) success, according to access type *.

	*MVS (Total)* *n = 517*	*FS vs. PS* *n = 243*	*p-Value*	*OR*	*CI*	*FS vs. MT* *n = 402*	*p-Value*	*OR*	*CI*
*Combined operative success—yes (%)*	89.0	85.2	0.155	0.60	0.29–1.22	91.0	0.188	1.60	0.80–3.22
*EuroSCORE2 adjusted*			0.162	0.60	0.29–1.23		0.373	1.39	0.67–2.84
*Combined perioperative success—yes (%)*	74.1	63.8	0.529	0.85	0.50–1.43	77.6	<0.001	2.60	1.60–4.21
*EuroSCORE2 adjusted*			0.470	0.82	0.48–1.40		0.002	2.19	1.32–3.63
*30-day survival (%)*	97.9	96.3	0.398	1.84	0.449–7.52	98.0	0.021	6.69	1.33–33.61
*MI (%)*	0.6	0.4	0.997	n.a		0.5	0.956	1.07	0.10–11.92
*ECMO (%)*	2.5	1.6	0.292	0.294	0.030–2.87	2.2	0.168	0.232	0.3–1.85
*Renal failure dialysis (%)*	7.9	15.6	0.079	0.531	0.26–1.08	4.5	<0.001	11.991	3.41–42.23
*>24 h ventilation (%)*	15.3	20.1	0.703	1.13	0.60–2.1	14.2	0.007	2.17	1.23–3.84
*Reoperation for any reason (%)*	7.4	8.6	0.668	1.22	0.49–3.0	4.2	0.256	1.56	0.72–3.38
*Reoperation bleeding (%)*	6.2	7.0	0.631	0.79	0.29–2.11	5.7	0.755	1.15	0.48–2.8
*PM implantation (%)*	3.5	7.0	0.329	0.607	0.223–1.652	2.0	0.01	15.79	1.9–129.8
*CVE (%)*	0.8	1.6	0.386	2.74	0.28–26.68	0	0.99		

Abbreviations: CI = confidence interval; CVE = cerebrovascular adverse event; ECMO = extracorporeal membrane oxygenation; FS = full sternotomy; MI = myocardial infarction; MVS = mitral valve surgery; OR = odds ratio; PM = pacemaker; * reference access is full sternotomy.

## Data Availability

Data is unavailable due to privacy or ethical restrictions.

## References

[1] Nkomo V.T., Gardin J.M., Skelton T.N., Gottdiener J.S., Scott C.G., Enriquez-Sarano M. (2006). Burden of valvular heart diseases: A population-based study. Lancet.

[2] O’Brien S.M., Shahian D.M., Filardo G., Ferraris V.A., Haan C.K., Rich J.B., Normand S.-L.T., DeLong E.R., Shewan C.M., Dokholyan R.S. (2009). The Society of Thoracic Surgeons 2008 cardiac surgery risk models: Part 2—Isolated valve surgery. Ann. Thorac. Surg..

[3] Grant S.W., Hickey G.L., Modi P., Hunter S., Akowuah E., Zacharias J. (2019). Propensity-matched analysis of minimally invasive approach versus sternotomy for mitral valve surgery. Heart.

[4] Kang D.H., Heo R., Lee S., Baek S., Kim D.-H., Song J.-M., Song J.-K., Lee J.W. (2018). Initial surgery versus conservative management of symptomatic severe mitral regurgitation in the elderly. Heart.

[5] Bonnet V., Boisselier C., Saplacan V., Belin A., Gérard J.-L., Fellahi J.-L., Hanouz J.-L., Fischer M.-O. (2016). The role of age and comorbidities in postoperative outcome of mitral valve repair: A propensity-matched study. Medicine.

[6] Seeburger J., Falk V., Garbade J., Noack T., Kiefer P., Vollroth M., Mohr F.W., Misfeld M. (2012). Mitral valve surgical procedures in the elderly. Ann. Thorac. Surg..

[7] Beckmann A., Meyer R., Lewandowski J., Markewitz A., Blassfeld D., Boning A. (2022). German Heart Surgery Report 2021: The Annual Updated Registry of the German Society for Thoracic and Cardiovascular Surgery. Thorac. Cardiovasc. Surg..

[8] Gotte J., Zittermann A., Hakim-Meibodi K., Hata M., Schramm R., Bleiziffer S., Parsa M.A., Gummert J., Renner A. (2022). Long-Term Clinical Outcome in Elderly Patients Undergoing Mitral Valve Repair. Thorac. Cardiovasc. Surg..

[9] Kawajiri H., Schaff H.V., Dearani J.A., Daly R.C., Greason K.L., Arghami A., Rowse P.G., Viehman J.K., Lahr B.D., Gallego-Navarro C. (2022). Clinical Outcomes of Mitral Valve Repair for Degenerative Mitral Regurgitation in Elderly Patients. Eur. J. Cardiothorac. Surg..

[10] Vollroth M., Seeburger J., Garbade J., Borger M.A., Misfeld M., Mohr F.W. (2013). Conversion rate and contraindications for minimally invasive mitral valve surgery. Ann. Cardiothorac. Surg..

[11] Doenst T., Diab M., Sponholz C., Bauer M., Farber G. (2017). The Opportunities and Limitations of Minimally Invasive Cardiac Surgery. Dtsch. Arztebl. Int..

[12] Cetinkaya A., Geier A., Bramlage K., Hein S., Bramlage P., Schönburg M., Choi Y., Richter M. (2021). Long-term results after mitral valve surgery using minimally invasive versus sternotomy approach: A propensity matched comparison of a large single-center series. BMC Cardiovasc. Disord..

[13] Zacharias J., Perier P. (2020). Seven Habits of Highly Effective Endoscopic Mitral Surgeons. Innovations.

[14] Oezpeker U.C., Barbieri F., Hoefer D., Bonaros N., Grimm M., Mueller L. (2022). Partial Upper Sternotomy is a Safe Alternative in Mitral Annulus Decalcification. Semin. Thorac. Cardiovasc. Surg..

[15] Hage A., Hage F., Al-Amodi H., Gupta S., Papatheodorou S.I., Hawkins R., Ailawadi G., Mittleman M.A., Chu M.W.A. (2021). Minimally Invasive versus Sternotomy for Mitral Surgery in the Elderly: A Systematic Review and Meta-Analysis. Innovations.

[16] Oezpeker C., Barbieri F., Hoefer D., Schneider B., Bonaros N., Grimm M., Mueller L. (2019). Mitral Valve Surgery via Partial Upper Sternotomy: Closing the Gap between Conventional Sternotomy and Right Lateral Minithoracotomy. Thorac. Cardiovasc. Surg..

[17] Bonaros N., Hoefer D., Oezpeker C., Gollmann-Tepeköylü C., Holfeld J., Dumfarth J., Kilo J., Ruttmann-Ulmer E., Hangler H., Grimm M. (2022). Predictors of safety and success in minimally invasive surgery for degenerative mitral disease. Eur. J. Cardio-Thorac. Surg..

[18] Gaur P., Kaneko T., McGurk S., Rawn J.D., Maloney A., Cohn L.H. (2014). Mitral valve repair versus replacement in the elderly: Short-term and long-term outcomes. J. Thorac. Cardiovasc. Surg..

[19] Farid S., Ladwiniec A., Hernandez-Sanchez J., Povey H., Caruana E., Ali A., Moorjani N., Irons J., Ring L., Abu-Omar Y. (2019). Early Outcomes after Mitral Valve Repair versus Replacement in the Elderly: A Propensity Matched Analysis. Heart Lung Circ..

[20] Bonaros N., Hofer D., Holfeld J., Grimm M., Muller L. (2020). Cannulation of the Carotid Artery for Minimally Invasive Mitral or Tricuspid Valve Surgery. Ann. Thorac. Surg..

[21] Al Otaibi A., Gupta S., Belley-Cote E.P., Alsagheir A., Spence J., Parry D., Whitlock R.P. (2017). Mini-thoracotomy vs. conventional sternotomy mitral valve surgery: A systematic review and meta-analysis. J. Cardiovasc. Surg..

[22] Oezpeker C.U., Barbieri F., Hoefer D., Bonaros N., Grimm M., Mueller L. (2022). Upper Hemi-Sternotomy Provides Benefit for Patients with Isolated or Combined Mitral Valve Surgery. Medicina.

[23] Vlahakes G.J. (2012). Right ventricular failure after cardiac surgery. Cardiol. Clin..

[24] Zanobini M., Loardi C., Poggio P., Tamborini G., Veglia F., di Minno A., Myasoedova V., Mammana L.F., Biondi R., Pepi M. (2018). The impact of pericardial approach and myocardial protection onto postoperative right ventricle function reduction. J. Cardiothorac. Surg..

[25] Chivasso P., Bruno V.D., Farid S., Malvindi P.G., Modi A., Benedetto U., Ciulli F., Abu-Omar Y., Caputo M., Angelini G.D. (2018). Predictors of survival in octogenarians after mitral valve surgery for degenerative disease: The Mitral Surgery in Octogenarians study. J. Thorac. Cardiovasc. Surg..

[26] Moscarelli M., Emmanuel S., Athanasiou T., Speziale G., Fattouch K., Casula R. (2016). The role of minimal access valve surgery in the elderly. A meta-analysis of observational studies. Int. J. Surg..

[27] Loulmet D.F., Ranganath N.K., Neragi-Miandoab S., Koeckert M.S., Galloway A.C., Grossi E.A. (2019). Advanced experience allows robotic mitral valve repair in the presence of extensive mitral annular calcification. J. Thorac. Cardiovasc. Surg..

[28] Helmers M.R., Shin M., Iyengar A., Arguelles G.R., Mays J., Han J.J., Patrick W., Altshuler P., Hargrove W.C., Atluri P. (2021). Permanent pacemaker implantation following mitral valve surgery: A retrospective cohort study of risk factors and long-term outcomes. Eur. J. Cardiothorac. Surg..

[29] Lukac P., Hjortdal V., Pedersen A.K., Jensen H.K., Mortensen P.T., Hansen P.S. (2006). The superior transseptal surgical approach to mitral valve creates slow conduction. Pacing Clin. Electrophysiol..

